# Research priorities for advanced HIV disease in Latin America and the Caribbean region: a modified Delphi study

**DOI:** 10.1002/jia2.70074

**Published:** 2026-01-16

**Authors:** Evelina Chapman, Omar Sued, Jorge O. Maia Barreto, Claudia P. Cortes, Brenda Crabtree Ramírez, José E. Vidal, Antonio Camiro‐Zúñiga, Freddy Perez

**Affiliations:** ^1^ Department of Communicable Diseases Prevention, Control and Elimination, Pan American Health Organization Washington DC USA; ^2^ Oswaldo Cruz Foundation Brasília Brazil; ^3^ Center for HIV AIDS Integral Research‐Chair Facultad de Medicina Universidad de Chile and Hospital Clinico San Borja Arriarán Santiago Chile; ^4^ Departamento de Infectología, Instituto Nacional de Ciencias Médicas y Nutrición Salvador Zubirán Mexico City Mexico; ^5^ Serviço de Extensão ao Atendimento de Pacientes HIV/Aids (SEAP), Departamento de Infectologia e Medicina Tropical Faculdade de Medicina Universidade de São Paulo São Paulo Brazil; ^6^ Departamento de Neurologia Instituto de Infectologia Emílio Ribas São Paulo Brazil; ^7^ Federal University of Health Sciences of Porto Alegre (UFCSPA) Porto Alegre Brazil

**Keywords:** acquired immunodeficiency syndrome, Caribbean region, Delphi technique, HIV, Latin America, opportunistic infections, research priorities, systematic review

## Abstract

**Introduction:**

Advanced HIV disease (AHD) remains a leading cause of mortality in Latin America and the Caribbean (LAC), driven by late diagnosis, treatment gaps and structural barriers, particularly among key populations and children. Persistent disparities in healthcare access, stigma, and limited health system capacity highlight the need for targeted research to improve AHD outcomes in the region.

**Methods:**

The modified Delphi process to prioritize AHD research questions in LAC was conducted between August and November 2024. Systematic reviews, expert consultations and two Delphi rounds involving 74 and 69 participants from 17 LAC countries assessed questions based on public health relevance, feasibility and equity. A subsequent in‐person workshop with 24 experts refined and validated the results, organizing the prioritized questions into short‐, medium‐ and long‐term priorities.

**Results:**

Seventy‐seven high‐priority research questions were identified, 60 focused on adults and 17 on children. These questions centred on opportunistic infections (OIs), HIV‐related cancers and health system interventions. Tuberculosis was the most frequently addressed OI (44% of OI‐related questions), followed by cryptococcosis, histoplasmosis and HIV‐related malignancies. Short‐term priorities included interventions to reduce late diagnosis, improve retention in care and strengthen health systems, particularly for vulnerable populations such as children, pregnant women and incarcerated individuals.

**Conclusions:**

This study presents a comprehensive research agenda for AHD in LAC, emphasizing interventions to address OIs, strengthen the health system and support at‐risk populations. The prioritized questions provide a roadmap for researchers, policymakers and funders to allocate resources effectively, ultimately improving AHD outcomes and reducing HIV‐related mortality. Strengthening regional collaboration and political commitment will be critical to translating research into actionable policies and interventions.

## 1 INTRODUCTION

Globally, 40.8 million people were living with HIV in 2024, including 2.5 million in Latin America and the Caribbean (LAC) [1]. While worldwide HIV‐related deaths have declined by over 50% since 2010, LAC achieved only a 28% reduction, falling short of its 2025 target by an estimated 25,000 deaths annually [1]. Advanced HIV disease (AHD), defined as a CD4 count below 200 cells/µl or an AHD‐defining condition, remains the leading cause of HIV‐related mortality in the region [2, 3]. Its persistence reflects late diagnosis, treatment gaps and structural inequities that undermine timely care and health‐system response.

Late HIV diagnosis affects roughly 30–39% of new cases in LAC [1]. Contributing factors include inequitable healthcare access, stigma and discrimination towards key populations (e.g. migrants, people who use drugs, vulnerable populations and men who have sex with men), which undermine testing and treatment uptake [4]. Political and economic instability often disrupts HIV programme funding and treatment continuity [5]. Other barriers include limited access to pre‐exposure prophylaxis, insufficient antenatal HIV testing [6] and weak regional coordination to support mobile and migrant populations [1, 5]. The COVID‐19 pandemic further exacerbated these vulnerabilities within already strained and fragmented healthcare systems [7].

Health research is pivotal to reversing trends. The World Health Organization (WHO) emphasizes that robust research enhances decision‐making, identifies public health priorities and improves service delivery to advance universal health coverage [8]. Research is crucial for AHD to clarify disease dynamics, improve diagnostics and treatment, and identify barriers to effective implementation. [9]. Integrating findings from epidemiological studies, clinical trials and operational research, interested‐holders [10] can refine treatment protocols and overcome barriers to care, ensuring that innovations reach those most in need [9, 11].

Setting research priorities directs scarce resources towards interventions with the highest potential impact. In Brazil, national research informed early diagnosis and Antiretroviral therapy (ART)‐expansion policies, improving survival and serving as a reference model for other countries [12]. In Peru, operational research on social and structural determinants of access to timely HIV care among key populations and indigenous communities informed community‐based testing and decentralized treatment models [13]. These experiences demonstrate the value of context‐specific evidence for policy transformation. However, no structured regional agenda has yet defined AHD research priorities in the LAC region.

This study aimed to identify and prioritize research questions on AHD across LAC using a modified Delphi methodology. The objective was to develop a comprehensive, consensus‐based regional research agenda to guide future studies, funding and policy actions, ultimately reducing HIV‐related mortality and improving health outcomes throughout the region.

## 2 METHODS

The research prioritization process was conducted between August and November 2024. The study protocol was reviewed by the Pan American Health Organization (PAHO) Ethics Committee and granted a research exemption (PAHOERC.0783.01‐exempt). Oversight was provided by a multidisciplinary Technical Advisory Group, with experts from four countries, including PAHO advisors and researchers (Table S1). The process followed WHO guidelines for systematic research priority setting [14].

All participants received written information about the study's objectives, procedures, data use, confidentiality safeguards and the voluntary nature of participation. Written informed consent was obtained electronically for both Delphi survey rounds. For the in‐person workshop, consent was implied through acceptance of the invitation as approved by the ethics committee.

### 2.1 Step 1: systematic reviews, searches and research gaps mapping

**Figure 1:**
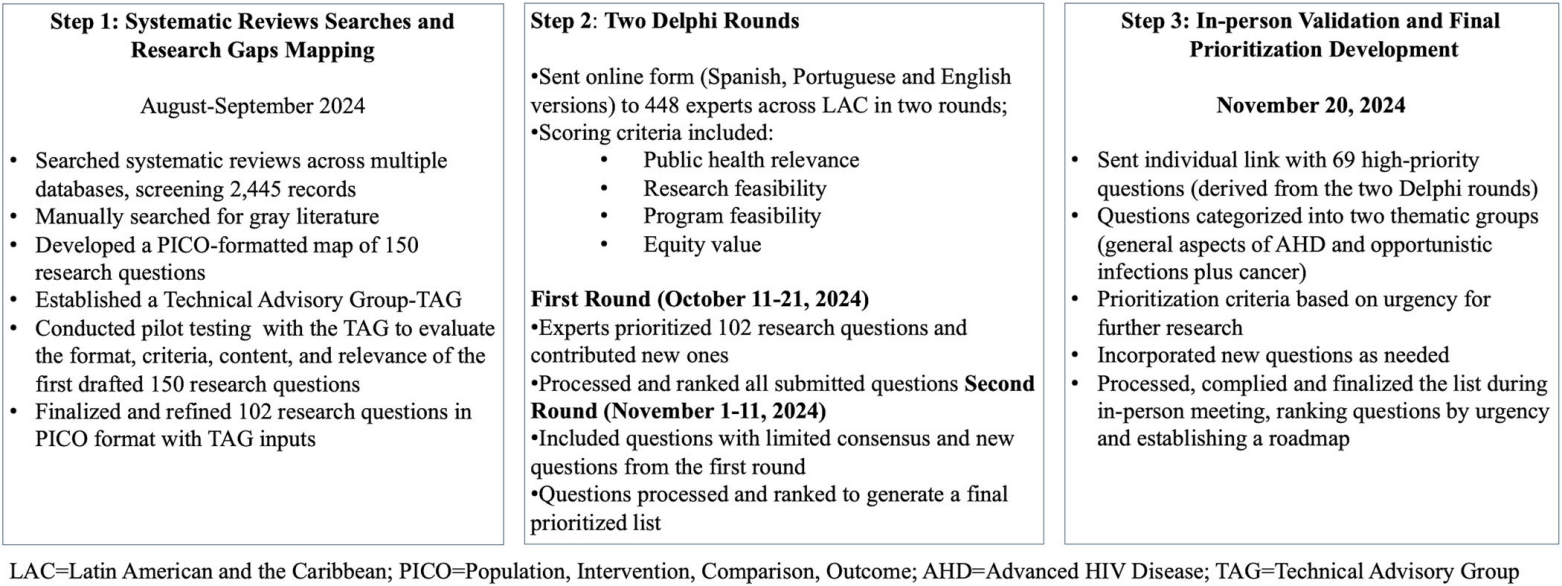
Process of setting research priorities for advanced HIV disease in Latin America and the Caribbean.

A mapping of research gaps was conducted through systematic reviews published in the past decade [15, 16, 17]. These reviews informed a preliminary list of questions, later prioritized by regional experts and interest‐holders using a modified Delphi methodology [18, 19], implemented in three steps (Figure 1). The framework aligned with the WHO research landscape on AHD, tailored to the LAC context over 10 years [20].

#### 2.1.1 Search strategy

A comprehensive search was conducted in electronic databases, including PubMed and the Virtual Health Library in English, Spanish and Portuguese, as well as grey literature sources (policy reports and conferences) (Google Scholar, PAHO/WHO websites) [21]. Guidelines using the GRADE methodology were manually searched in the Base Internacional de Guias GRADE database within the Virtual Health Library. Expert consultation complemented the literature review. PubMed searches were performed on 25 August 2024.

#### 2.1.2 Search terms

Both controlled vocabulary and free‐text terms were used. The DeCS/MeSH database (https://decs.bvsalud.org/) helped identify terms in Portuguese and Spanish. The complete search strategy is provided in Table S2.

#### 2.1.3 Eligibility criteria

Systematic reviews were included if they addressed any of the following in LAC: children under 5 with HIV, AHD mortality risk factors, late diagnosis interventions, AHD diagnostics, associated infections or malignancies, treatment adherence strategies, patient re‐engagement and palliative care. Studies focusing on non‐HIV comorbidities, viral hepatitis, antiretroviral (ARV) drug development and HIV‐drug resistance were excluded.

#### 2.1.4 Selection process

Two independent reviewers (EC and JOMB) screened 2445 systematic reviews. Duplicates were removed using Rayyan®, an online tool for managing systematic reviews. After screening titles and abstracts, 361 reviews were selected for full‐text evaluation by consensus.

#### 2.1.5 Pilot assessment and research question development

Research gaps were synthesized into 150 Population, Intervention, Comparison, Outcome‐formatted questions using the selected systematic reviews and grey literature. A pilot assessment by the Technical Advisory Group (TAG) evaluated the format, criteria, content and relevance of the initial 150 drafted questions. Reviews published before 2018 were deprioritized due to outdated evidence, narrowing the focus to reviews from 2019 to 2024. Additional research questions were generated based on knowledge gaps identified in the WHO's 2021 consolidated HIV guidelines [3].

Questions were categorized by population and topic. For children under 5, topics included general aspects of advanced HIV, opportunistic infections (OIs) and the transition from childhood to adolescence. For adults, cancer‐related questions in the context of HIV and AHD were also included.

### 2.2 Step 2: two Delphi rounds

#### 2.2.1 First round: survey development and implementation

A structured survey was designed using Google Forms®, initially developed in Spanish and later translated into English and Portuguese by native speakers. It consisted of two sections: demographic and professional background, and prioritization of research questions. Four predefined prioritization criteria, adapted from Gottlieb et al. [22], were used: (1) public health relevance; (2) research feasibility; (3) programme feasibility; and (4) equity value. Each criterion was scored on a 5‐point Likert scale from “Strongly disagree” to “Strongly agree.” Respondents could propose new Population, Intervention, Comparison, Outcome questions and provide comments.

Experts were selected from a PAHO‐established regional panel with diverse AHD expertise. Personal invitations were sent via official PAHO email to 448 experts on 11 October 2024, with a 10‐day response window and three reminders.

#### 2.2.2 First round analysis

Survey data were compiled in Excel, and a descriptive analysis was conducted based on demographic data. For prioritization, the frequency of each Likert scale response was calculated. A priority index was computed for each question and criterion, measuring the proportion of “Agree” and “Strongly Agree” responses out of the total responses: (*n* “Agree” + *n* “Strongly Agree”)/total responses, ranging from 0 to 1. The average across the four criteria was used to calculate an aggregate prioritization index.

Questions with an aggregate index of ≥0.80 were classified as “high priority.” Those with ≤0.69 were excluded. Questions scoring between 0.70 and 0.79 were reconsidered in Round 2 due to insufficient consensus (Table S3).

#### 2.2.3 Second round development of survey and participants

The second survey included unresolved questions from the first round and new submissions from participants. The Technical Advisory Group reviewed all items to remove duplicates and ensure consistency. Highly priority questions from Round 1 (index ≥ 0.80) were not re‐evaluated. A second Google Forms® survey was developed in Spanish, English and Portuguese, maintaining the same structure as the first round and including a consent to participate. The four prioritization criteria remained unchanged, but the 5‐level Likert scale was revised from “Agree/Disagree” to “Not a priority” to “High priority.” The survey was distributed to the same 448 experts on 1 November 2024, with a 10‐day response window and three reminders.

#### 2.2.4 Second round analysis

The analysis followed the same process as Round 1. Questions with an aggregate index ≥0.80 were deemed “high priority” (Table S4).

### 2.3 Step 3: in‐person validation and final prioritization

An in‐person validation workshop was held in Bogotá, Colombia, on 20 November 2024, with HIV experts from 12 countries (Argentina, Brazil, Chile, Colombia, Cuba, Ecuador, El Salvador, Guatemala, Paraguay, the United States, Uruguay and the United Kingdom) and PAHO staff. The meeting aimed to validate, refine and reprioritize research questions and classify them by time frame: short‐term (<2 years), medium‐term (2−4 years) and long‐term (>4 years), reflecting both feasibility and the expected timeline for generating actionable evidence. A 5‐year implementation horizon was adopted. Activities included:

1. *Individual prioritization*: Participants independently ranked high‐priority questions from previous rounds by urgency (short‐term, medium‐term or long‐term) via an online form (Google Forms®). Real‐time frequencies were presented for each category to inform the discussion.
2. *Group discussions and new question development*: Experts were divided into four thematic groups, two focused on general aspects of AHD and two on OIs and malignancies. Each group reviewed prioritization results, generated new Population, Intervention, Comparison, Outcome‐format questions and classified questions by urgency level.
3. *Final plenary consensus session*: A final session validated the 5‐year research agenda and discussed dissemination strategies to facilitate uptake by regional and national interested‐holders.

## 3 RESULTS

### 3.1 First Delphi round

Conducted from 11 to 21 October 2024, the first Delphi round evaluated 102 research questions. Seventy‐four experts from 17 countries participated, yielding a response rate of 16.5%. Most were from Mexico, Brazil, Argentina and Chile, accounting for 60% of respondents. One participant opted out and was excluded thereafter.

Among the 74 participants, 54% identified as cisgender men (*n* = 40), 44.6% as cisgender women (*n* = 33) and one as non‐binary. Most (77%) were under 54. Professional backgrounds included medicine (36.5%), research (28.4%), academia (11%) and activism (4%). The median HIV‐related experience was 15 years. Participants were employed in hospitals/clinics (31%), national or regional governments (29.7%) and academic/research institutions (20.3%).

Of the 102 research questions assessed, 58 scored as high priority (aggregate index ≥0.80) and were reserved for discussion during the in‐person meeting. Thirty questions that did not achieve a strong consensus (index between 0.70 and 0.79) were advanced to the second Delphi round, along with 14 new questions proposed by participants. Fourteen questions were ranked as low priority (index ≤0.69) and excluded (Table S3).

### 3.2 Second Delphi round

Held from 1 to 11 November 2024, this round evaluated 44 research questions. Sixty‐nine experts from 17 countries participated, yielding a response rate of 15.4%. Nearly half were from Brazil, Chile, Peru and Mexico. Among respondents, 38 identified as cisgender men (55%) and 31 as cisgender women (45%). Demographics and professional profiles mirrored Round 1. No significant differences were noted overall (Table S5). Of the 44 questions, 10 reached high‐priority status (index ≥0.80) (Table S4). In total, 69 high‐priority questions advanced to final validation.

### 3.3 In‐person validation and final prioritization

Twenty‐four professionals from 12 countries (as detailed in the Methods section) participated in the final in‐person validation. The group included 19 physicians, one nurse, three researchers and one biochemist, all HIV experts. During the session, 15 new questions were added, and seven previously selected were removed, resulting in 77 priority research questions (Table 1).

**Table 1:** Prioritized research questions on advanced HIV disease categorized by age group, themes, subthemes and level of urgency for response

Short‐term questions
**Adults Opportunistic infections Diagnosis** What is the performance of the concomitant use of Xpert on sputum and TB‐LAM on urine for diagnosing Mycobacterium tuberculosis infection in patients with advanced HIV disease? What is the impact on mortality of combined diagnostic interventions such as urine LAM, cryptococcal antigen tests, rapid CD4 count and viral load measurements in people on antiretroviral therapy who are hospitalized in Latin America and the Caribbean? What is the performance of advanced versions of the QuantiFERON‐TB assay (QFT‐plus) for detecting latent tuberculosis infection in patients with advanced HIV disease? Is it cost‐effective to include a rapid molecular test and establish a cut‐off point to differentiate between active disease and colonization in pharyngeal swab/wash/saliva at point‐of‐care for managing pneumocystosis in individuals with advanced HIV disease? What is the appropriate CD4 cut‐off point for screening for opportunistic infections in advanced HIV disease in Latin America and the Caribbean and/or symptomatic individuals? Can machine learning improve the reading of rapid tests for opportunistic infections in advanced HIV disease? **Frequency** What is the prevalence of histoplasmosis in individuals with advanced HIV disease (measured by antigenuria) in Latin America and the Caribbean? What is the prevalence of disseminated histoplasmosis in adolescents and adults with advanced HIV disease? What is the prevalence of cryptococcosis in individuals with advanced HIV disease in Latin America and the Caribbean? What is the prevalence of cryptococcal antigenemia among individuals living with HIV who have CD4 counts < 200/<100 in Latin America and the Caribbean? **Risk factor** What are the most significant and modifiable risk factors contributing to tuberculosis‐associated mortality in individuals living with HIV? **Disease management** What is the impact of cryptococcal antigen (CrAg) detection and preventive fluconazole therapy on the incidence of cryptococcal meningitis among adults living with HIV who have CD4 counts <100 cells/µl and have not previously received antiretroviral therapy? What are the best available treatment options for individuals living with HIV co‐infected with tuberculosis and histoplasmosis, given the contraindication of concomitant use of itraconazole and first‐line tuberculosis therapies (e.g. isoniazid, rifampin, pyrazinamide and ethambutol)? What is the appropriate management for asymptomatic individuals with advanced HIV disease who have a positive urine histoplasma antigen (HAg) test? **Health systems and services** What role do community‐led, virtual and differentiated service delivery models play in addressing the comorbidity of tuberculosis and HIV in Latin America and the Caribbean? **Policies** What comprehensive strategies can reduce tuberculosis treatment dropout rates among vulnerable groups with advanced HIV disease (e.g. homeless individuals, men who have sex with men, among others)? **General aspects of advanced HIV disease Diagnosis** What is the cost‐effectiveness of HIV‐1 RNA assays at the point of care in the Latin America and the Caribbean region? **Risk factors** What is the impact of poverty and deprivation on disease progression and mortality among individuals with advanced HIV disease? **Health systems and services** What is the prevalence of late initiation of antiretroviral therapy among individuals newly diagnosed with advanced HIV disease in Latin America and the Caribbean? What effective strategies improve retention in prenatal and/or postpartum care among adolescent and young women living with HIV? What are the barriers to the early initiation of antiretroviral therapy in newly diagnosed individuals with advanced HIV disease? What are the barriers and facilitators of interventions aimed at improving linkage to healthcare services for individuals with advanced HIV disease?
What interventions can ensure proper linkage and continuity of support following hospital discharge for patients with advanced HIV disease to reduce mortality? **Policies** What gaps at the patients, providers and healthcare system levels contribute to loss to follow‐up among patients living with HIV in Latin America and the Caribbean? What HIV prevention and treatment strategies are effective in institutions housing incarcerated individuals in Latin America and the Caribbean? What key indicators of advanced HIV disease are relevant for the Latin America and the Caribbean region, and how can they be standardized? What strategies ensure the sustainable implementation of rapid diagnostic testing management? **Cancer Diagnosis** In men who have sex with men (MSM) with advanced HIV disease, how can PCR be used for early detection of HPV and prevention of anal cancer? **Disease management** What is the impact of the HPV vaccine on individuals with advanced HIV disease and HPV‐related lesions in Latin America and the Caribbean?
**Children Opportunistic infections Diagnosis** What are the optimal tuberculosis screening strategies for children living with HIV in Latin America and the Caribbean? What is the cost‐effectiveness of modern tuberculosis diagnostic tests (such as Fuji‐LAM, Alere LAM, IGRA) for detecting co‐infection in children living with HIV? **Frequency** What are the incidence and prevalence of opportunistic infections in children living with HIV in Latin America and the Caribbean? What are the mortality rate and risk factors for children and adolescents co‐infected with HIV and tuberculosis in Latin America and the Caribbean? **Disease management** What is the appropriate management for asymptomatic children with advanced HIV disease who test positive for TB LAM? **Health systems and services** What is the availability and accessibility of rapid diagnostic tests for children with advanced HIV disease? and young women living with HIV? **Policies** What is the frequency of preventive tuberculosis treatment implementation in children under 5 years of age living with HIV? **General aspects of advanced HIV disease Frequency** What is the prevalence of advanced HIV disease among adolescents transitioning from paediatric services to adult healthcare services in Latin America and the Caribbean? **Health systems and services** What strategies effectively ensure retention in antiretroviral treatment and minimize premature mortality among children living with HIV in Latin America and the Caribbean? What interventions support continuity of care and prevent disease progression among people living with HIV during the transition from paediatric to adult care in Latin America and the Caribbean? What is the magnitude of dropout from early diagnostic services among children exposed to HIV in Latin America and the Caribbean? What interventions effectively increase early HIV diagnosis in infants exposed to the virus within the first 4–8 weeks of life? How does the transition from paediatric to adult care impact the continuity of HIV care in Latin America and the Caribbean?
**Medium‐term questions**
**Adults Opportunistic infections Diagnosis** What is the effectiveness of PCR using a swab versus an oral wash in guiding the management of *Pneumocystis jirovecii* pneumonia in individuals with advanced HIV disease? **Frequency** What is the frequency of *Toxoplasma gondii* reactivation in individuals living with HIV in Latin America and the Caribbean? What is the frequency of *Mycobacterium avium* complex disease in individuals with advanced HIV disease? What is the morbidity and mortality burden of nosocomial infections in hospitalized individuals with advanced HIV disease?
**Risk factor** What factors contribute to a higher risk of acquired rifamycin resistance during first‐line treatment for TB in advanced HIV disease? What role does advanced HIV disease play in therapeutic failures of multiple‐drug‐resistant tuberculosis (TB)? **Disease management** What is the efficacy and safety of regimens for the prevention of cryptococcal disease in individuals living with HIV and positive cryptococcal antigenemia in plasma or peripheral blood in Latin America and the Caribbean? What is the efficacy and safety of induction regimens for the treatment of cryptococcal meningitis in people living with HIV in Latin America and the Caribbean? What are the best strategies for clinical suspicion and screening of *Mycobacterium avium* complex (MAC) disease in individuals living with HIV? **Policies** In medium‐ or low‐resource settings, is it better to centralize or decentralize laboratories to meet the demand for diagnostic testing for opportunistic infections in individuals with advanced HIV disease? What community‐based intervention strategies could effectively improve the availability and access to rapid diagnostic tests for opportunistic infections in individuals with advanced HIV disease? **General aspects of advanced HIV disease Frequency** What are the causes of death in individuals with advanced HIV disease 1 year after diagnosis and initiation of treatment? **Disease management** What is the impact of counselling for patients with advanced HIV disease and poor adherence to treatment on re‐engagement to initial antiretroviral therapy and achieving viral re‐suppression? **Health systems and services** Are psychosocial interventions integrated into care programmes for advanced HIV disease effective and cost‐effective in reducing severe morbidity or mortality in the Latin America and the Caribbean region? What specific interventions improve retention in and access to HIV care services in intravenous drug users? **Policies** How can the experiences and perspectives of indigenous peoples/original communities in Latin America and the Caribbean be integrated into HIV care to improve access to culturally adapted and safe medical services? What socio‐demographic variables (e.g. racial and ethnic minorities) influence health literacy in individuals living with HIV, and how do they affect adherence to antiretroviral treatment and utilization of health services? Are integrated service delivery models (e.g. HIV, tuberculosis, substance abuse treatment, prevention of mother‐to‐child transmission programmes and mental healthcare for HIV) effective for retaining or re‐linking patients with advanced HIV disease to antiretroviral therapy? What are the best strategies for engaging and re‐engaging migrant populations, including pregnant women, with HIV care services? **Cancer Frequency** What is the prevalence of lymphoproliferative diseases, including primary central nervous system lymphoma, in patients with advanced HIV disease in Latin America and the Caribbean? **Disease management** Given that cervical cancer in women with HIV tends to present in more advanced stages compared to HIV‐negative women, what diagnostic, treatment and follow‐up strategies could improve quality of life and reduce mortality in Latin America and the Caribbean?
**Children General aspects of advanced HIV disease Risk factors** What factors contribute to high rates of loss to follow‐up among children under 5 years of age with HIV in Latin America and the Caribbean? **Health systems and services** What are the effects of integrating HIV testing into immunization consultations for children to increase early diagnosis? **Policies** What strategies for scaling up community‐based primary healthcare interventions are effective in improving HIV‐related health outcomes among the maternal and child population in the context of Latin America and the Caribbean? How can adherence to antiretroviral treatment be improved in children and adolescents living with HIV, particularly among those who are double orphans?
**Long‐term questions**
**Adults Opportunistic infections Diagnosis** What is the PCR cut‐off point for differentiating between *Pneumocystis jirovecii* pneumonia and colonization in individuals with advanced HIV disease?
**Disease management** Is the 3‐month isoniazid and rifapentine regimen (3HP), cost‐effective for treating latent tuberculosis in patients with advanced HIV disease? How does the efficacy and safety of preventive isoniazid treatment for tuberculosis vary among different sub‐populations of people living with HIV in Latin America and the Caribbean, considering diverse health determinants? What are the clinical outcomes and efficacy of amphotericin B treatment in pregnant women living with HIV who have cryptococcal meningitis? **General aspects of advanced HIV disease Health systems and services** What is the effect of patient navigation on individuals with advanced HIV disease across different population groups, and how does it impact outcomes in the continuum of HIV care (e.g. re‐linkage, retention)? Is the combination of reminder/alarm devices with other strategies, such as counselling, cost‐effective and acceptable for improving medication adherence in pregnant women living with HIV in Latin America and the Caribbean? What is the effectiveness of text messaging through mobile phones for improving adherence to antiretroviral therapy in adolescents in Latin America and the Caribbean? What adherence assessment tools for antiretroviral medications are effective in predicting the risk of treatment failure in individuals with advanced HIV disease? **Policies** What is the impact of peer woman accompaniment programmes on linkage to care after diagnosis in late‐stage disease and retention in care among newly diagnosed cisgender women? **Cancer Frequency** What is the prevalence of Kaposi's sarcoma in individuals with advanced HIV disease in Latin America and the Caribbean?

Abbreviations: AHD, advanced HIV disease; LAC, Latin American and the Caribbean; PICO, Population, Intervention, Comparison, Outcome; TAG, Technical Advisory Group.

Of the 77 prioritized research questions, 17 focused on children; of these, 10 addressed general aspects, and seven addressed specific OIs. The remaining 60 questions focused on adults, including 24 on general aspects, 31 on OIs and five on cancer. Overall, 42 were classified as requiring a short‐term response, 57% relating to specific OIs (Table 1).

Among pathogens, tuberculosis (TB) was the most frequently cited pathogen, appearing in 17 of 38 OIs‐related questions (44.7%), followed by *Cryptococcus* spp. and *Histoplasma* spp. Others included *Pneumocystis jirovecii*, *Mycobacterium avium* complex, human papillomavirus (HPV) and *Toxoplasma gondii*. Notably, all OI‐related questions for children were classified as urgent. Five focused on HIV‐related cancers: two short‐term questions on HPV‐related cancers (anal and cervical), two medium‐term questions on the frequency of lymphoproliferative diseases and cervical cancer management, and one long‐term question on the regional prevalence of Kaposi's sarcoma. No questions addressed cancer in children.

Short‐term priorities emphasized health systems interventions (10 of 17) and policy‐related issues (4 of 17). Medium‐term priorities were predominantly policy‐focused (6 out of 12), followed by health systems and service delivery (3 of 12). Long‐term priorities were also largely policy‐oriented (4 of 5). The complete list of research questions is presented in Table 1 with thematic and temporal breakdowns, and in Table 2, a synthesis.

**Table 2:** Synthesis of final prioritized research questions on AHD categorized by age group, themes and subthemes (*n* = 77)

Topic/Sub‐topic	
Adults	*n*
*Opportunistic infections (31)*	
Tuberculosis	9
Cryptococcosis	6
Histoplasmosis	3
*Mycobacterium avium* complex disease	2
*Pneumocystis jirovecii* pneumonia	3
Tuberculosis and histoplasmosis	1
Tuberculosis and cryptococcosis	1
Toxoplasmosis	1
Others^a^	5
*General aspects of advanced HIV disease (24)*	
Engagement/re‐engagement with services	9
Adherence to treatment	5
HIV mortality	2
Early initiation of ARVs	2
Psychosocial interventions	2
Sustainable rapid tests	1
Prevention and treatment (prisoners)	1
Cost‐effectiveness of tests (point‐of‐care)	1
Standardization of indicators	1
Access to indigenous populations	1
*Cancer (5)*	
Papillomavirus‐associated cancer (anal and cervical)	2
Cervical cancer	1
Kaposi's sarcoma	1
Lymphoproliferative diseases	1
**Children**	
*Opportunistic infections (7)*	
Tuberculosis	6
Others^a^	1
*General aspects of advanced HIV disease (10)*	
Early diagnosis	3
Continuity of care (childhood/adolescence)	3
Loss of follow‐up (under 5 years)	2
Treatment adherence in orphans	1
Scaling up primary care (maternal‐infant)	1
**Total**	**77**

^a^Others in adults: HIV opportunistic infection screening, machine learning for rapid test interpretation, nosocomial infection burden, laboratory centralization versus decentralization and community‐based diagnostic access. In children: frequency of OIs.

### 3.4 Roadmap for dissemination and implementation

Key recommendations emphasized strengthening the regional research ecosystem through coordinated networks of researchers, academic partnerships, national research agencies, community organizations and ministries of health, together with support from international funding agencies. Proposed dissemination strategies included multi‐format materials, a digital platform and tailored communications to engage policymakers, researchers and affected communities.

Integrating the research agenda into national policies was considered essential, requiring sustained engagement with ministries of health and civil society. To ensure long‐term viability, participants recommended securing sustainable funding through a combination of international donors and regional investments.

A coordinated regional approach, under PAHO's leadership, was proposed to align country efforts, facilitate multicentre studies and promote harmonized implementation strategies. The roadmap also emphasized the importance of initiating short‐term actions with measurable outcomes to generate early impact and build momentum for long‐term reforms and innovative care models.

## 4 DISCUSSION

This study systematically identified and prioritized 77 high‐priority research questions on AHD in LAC using a modified Delphi method. Questions were categorized by urgency (short‐, medium‐ and long‐term) and grouped into thematic areas: general AHD aspects, OIs and HIV‐related cancers.

OIs accounted for most questions, reflecting their continued high burden in LAC [23, 24]. Recent studies report persistently high rates of late‐stage OIs and associated mortality [25], particularly for TB, oesophageal candidiasis, and *Pneumocystis jirovecii* pneumonia [26]. TB was the most frequently cited OI, included in 44% of all OI‐related questions and 86% of paediatric‐focused questions. Notably, 59% of TB‐related questions were short‐term priorities, aligned with TB's role as the leading cause of AHD‐related mortality in the region. A regional study linked TB co‐infection to increased long‐term mortality [27], and WHO estimated 10, 000 TB‐related deaths in 2023, representing one‐third of all AHD‐related deaths in LAC [28].

Prioritized TB‐related questions focused on expanding access to newer diagnostics and treatments (e.g. TB‐lipoarabinomannan Point‐of‐Care (POC) tests, tuberculosis preventive treatment [TPT] with rifapentine and QuantiFERON‐TB Gold Plus), improving screening rates and TPT coverage among people with HIV, and systemic vulnerabilities contributing to high mortality rates in HIV/TB coinfection. Experts noted that scalable, cost‐effective interventions, particularly TB prevention and early detection of active disease, significantly reduce mortality [29]. With regional TPT coverage at 29% [28] and over half of TB cases diagnosed during HIV treatment initiation, integrated care strategies remain crucial [30].

Other high‐priority OIs included cryptococcosis, histoplasmosis and *Mycobacterium avium* complex disease. As with TB, most questions emphasized improved diagnostics (antigen detection for cryptococcosis and histoplasmosis, and PCR for pneumocystosis) and optimized management. Concerns included POC test cutoff points, management of asymptomatic infections, screening criteria, cryptococcal meningitis treatment efficacy and management of TB/histoplasma coinfection [31]. Experts also noted insufficient epidemiological data and weak surveillance for both OIs and AHD‐related cancers. Studies from Guatemala, Paraguay, and Trinidad and Tobago reported OIs in 25–38% of AHD cases, with high positivity for *Cryptococcus* spp. and *Histoplasma* spp. [23, 24, 32]. Modelling suggests cryptococcal meningitis accounts for nearly 20% of HIV‐related deaths in LAC [33], while disseminated histoplasmosis may exceed TB mortality among People living with HIV (PLWH) [34].

HIV‐associated malignancies were underrepresented in the prioritized questions. Only three focused on HPV‐related cancers, screening in men who have sex with men and cervical cancer management. Experts emphasized the need for stronger prevention measures and surveillance for HIV‐associated lymphoma and Kaposi's sarcoma, both still neglected despite high mortality [35].

Beyond OIs, gaps in health systems and policy implementation research were evident. Most short‐term priorities (85.3%) focused on late HIV presentation, poor retention in care and systemic barriers, consistent with studies documenting low ART uptake, high loss to follow‐up and suboptimal care retention [36]. For example, a Trinidad and Tobago study found 75% of AHD patients were lost to follow‐up before initiating care [24]. Additional urgent questions addressed care retention among children and adolescents, HIV prevention in incarcerated populations, poverty's impact and continuity of care for pregnant and postpartum women. A 2025 meta‐analysis reported 4% HIV prevalence in LAC prisons, emphasizing the need for early HIV detection and linkage to care in high‐risk populations [37]. High perinatal HIV transmission persists with delayed diagnosis among pregnant women [38]. Experts called for research on improving care delivery and retention across vulnerable groups.

Overall, 68% of prioritized questions focused on vulnerable populations, including children and adolescents, pregnant women, people who inject drugs, indigenous communities, migrants, people in poverty or homelessness, incarcerated individuals and racial/ethnic minorities. Socio‐economic inequalities in LAC heighten HIV risk due to limited awareness, restricted services access and constrained prevention [39]. A recent study showed that eliminating poverty in LAC by 2030 could reduce AHD incidence by 4–14%, underscoring the importance of targeting social determinants of health [40].

A smaller subset of questions addressed HIV monitoring and data systems, including POC technologies for viral load and CD4 testing, and the need for standardized AHD reporting to improve surveillance. Participants highlighted the potential of task‐shifting and integrating AHD care into primary health services, strategies validated in sub‐Saharan Africa [41] and considered cost‐effective for scaling HIV/OIs testing in low‐resource settings [42].

Limitations should be noted. Research prioritization is dynamic and requires periodic reassessment [8, 14]. While the Delphi method captures expert consensus, it may reflect selection bias. The moderate response rate (17−47%) could have influenced results. To mitigate these effects, the study used systematic reviews to inform initial questions [16, 17], and multiple feedback rounds ensured validation.

Several prioritized questions are immediately actionable given recent scientific and programmatic advances. In the short term, studies focused on integrated POC diagnostics for OIs (TB‐LAM, CrAg Lateral Flow Assay, histoplasmosis Lateral Flow Assay, multiplex molecular platforms) are particularly feasible due to WHO prequalification, reduced costs and expanding regional laboratory capacity [43, 44, 45]. Similarly, research on simplified AHD care packages and differentiated service delivery models can leverage on ongoing ART scale‐up and community‐based care initiatives supported by global and regional partners [46, 47]. Implementation studies evaluating screen‐and‐treat algorithms for histoplasmosis and cryptococcosis are also technically ready, as standardized protocols and rapid tests are now available across multiple LAC countries [23, 24, 32, 48]. Conversely, long‐term priorities, including the development of new anti‐fungal agents, host‐directed therapies and large‐scale data integration for real‐time surveillance, will require substantial investment and coordination [49, 50, 51]. Distinguishing short from long‐term priorities provides a practical roadmap linking scientific readiness to policy and funding opportunities, accelerating translation from research to impact.

While different prioritization frameworks balance broad interest‐holders engagement with expert panels [52], our approach effectively combined diverse regional expertise through two Delphi rounds and an in‐person validation meeting to align with key public health priorities [53]. Engaging diverse interest‐holders enhanced the legitimacy and practical relevance of the prioritization process, ensuring that identified research gaps reflect real programmatic needs. This inclusive approach aligns research efforts to strengthen HIV control and improve outcomes for people with AHDs.

Moving forward, the main challenge lies in translating this agenda into sustained, adequately funded research and implementation. The cost of inaction, continued late HIV diagnosis, recurrent OIs and preventable deaths will perpetuate both human and economic losses [54]. Conversely, investing in AHD research and innovation offers high returns through reduced mortality, fewer hospitalizations and stronger health systems [55]. Potential financing could come from national governments, PAHO, UNAIDS, regional development banks such as the Inter‐American Development Bank and the World Bank, as well as global health mechanisms such as the Global Fund and UNITAID, which support innovation and implementation research [56, 57, 58, 59]. Additional opportunities exist through bilateral and development cooperation agencies (e.g. the French Development Agency [AFD], Deutsche Gesellschaft für Internationale Zusammenarbeit (GIZ) GmbH, the German Agency for International Cooperation, the Canada's International Development Research Centre [IDRC]) and philanthropic partners (such as the Bill and Melinda Gates Foundation, the Wellcome Trust or the Clinton Health Access Initiative [CHAI]) [60, 61, 62]. Engagement of national science and technology agencies, including Brazil's National Council for Scientific and Technological Development (CNPq) and the National Council of Science and Technology (CONACYT), Mexico's national research and innovation funding agency, can secure co‐funding and long‐term sustainability through local ownership [63, 64]. Coordinating these actors and aligning their investments with the identified priorities provides a pragmatic and cost‐effective path to accelerate impact and prevent AHD from remaining a neglected aspect of the HIV response in LAC [65].

To ensure uptake, the roadmap will be shared through PAHO's regional HIV and OI networks, Ministries of Health, and academic and research consortia across LAC. These platforms can integrate the priorities into ongoing initiatives and national plans, linking them to capacity‐building and implementation studies, and promoting regional ownership and sustainability.

## 5 CONCLUSIONS

Strengthening political commitment, community engagement and regional collaboration will be essential to advance AHD research in LAC, particularly amid increasing political instability and declining global health financing. Governments, academic institutions and international organizations must coordinate efforts to ensure that investments in HIV research translate to tangible improvements in care, health outcomes and policy development across the region.

## FUNDING

This study was partially funded by the United Nations International Drug Purchase Facility—UNITAID (Grant No. 609002) (OS and FP received the award). PAHO supported and coordinated this work. PAHO staff participated in the design of the research prioritization process, data collection, analysis, interpretation and manuscript writing. EC and JOMB were consultants funded by PAHO‐WHO for this project. The funders had no role in study design, data collection and analysis, decision to publish or manuscript preparation.

## AUTHOR CONTRIBUTIONS

EC, JOMB, FP, OS and AC‐Z conceptualized and coordinated the research prioritization process, developed the protocol and oversaw survey implementation. EC and JOMB conducted electronic searches, independently selected the systematic reviews, developed the initial map of 150 research questions in PICO format and designed the trilingual surveys for both Delphi rounds. FP, OS, AC‐Z, CPC, BCR and JEV refined the questions and added new ones. JOMB conducted data analysis and, in collaboration with EC, carried out data curation, analysis, categorization and interpretation. EC, JOMB and FP drafted the initial manuscript, while all authors (OS, AC‐Z, CPC, BCR and JEV) made significant contributions to the subsequent reviews and gave their final consent to the publication of the final version. FP was ultimately responsible for submitting the manuscript for publication as part of the PAHO research prioritization working group.

## COMPETING INTERESTS

All authors declare no conflicts of interest.

## Supporting information




**Table S1**: Composition of the PAHO Regional Technical Advisory Group on advanced HIV disease in Latin America and the Caribbean.
**Table S2**: PubMed search strategy for systematic reviews on advanced HIV disease (25 August 2024).
**Table S3**: Research priorities for advanced HIV disease in Latin America and the Caribbean region, prioritized questions. (Survey 1, *n* = 102 questions).
**Table S4**: Research priorities for advanced HIV disease in Latin America and the Caribbean region, prioritized questions. (Survey 2, *n* = 44 questions).
**Table S5**: Setting research priorities for advanced HIV disease in Latin America and the Caribbean, participant characteristics (Delphi rounds 1 and 2).

## Data Availability

All data used for this study are available upon successful application to the study team via the corresponding author.
