# Supplementary material for: Research priorities for advanced HIV disease in Latin America and the Caribbean region: a modified Delphi study
Source: J Int AIDS Soc. 2026 Jan 16;29(1):e70074. doi: 10.1002/jia2.70074 (PMC12817252; doi:10.1002/jia2.70074)
Supplement: Supplementary file 1 — Table S1: Composition of the PAHO Regional Technical Advisory Group on advanced HIV disease in Latin America and the Caribbean. Table S2: PubMed search strategy for systematic reviews on advanced HIV disease (25 August 2024). Table S3: Research priorities for advanced HIV disease in Latin America and the Caribbean region, prioritized questions. (Survey 1, n = 102 questions). Table S4: Research priorities for advanced HIV disease in Latin America and the Caribbean region, prioritized questions. (Survey 2, n = 44 questions). Table S5: Setting research priorities for advanced HIV disease in Latin America and the Caribbean, participant characteristics (Delphi rounds 1 and 2). [file JIA2-29-e70074-s001.docx]

**Supporting Files**

**Table S1.** Composition of the PAHO Regional Technical Advisory Group on Advanced HIV Disease in Latin America and the Caribbean

**Table S2**. PubMed Search Strategy for Systematic Reviews on Advanced HIV Disease (August 25, 2024)

**Table S3.** Research Priorities for Advanced HIV Disease in Latin America and the Caribbean Region, Prioritized Questions (Delphi round 1, n=102 questions)

**Table S4.** Research Priorities for Advanced HIV Disease in Latin America and the Caribbean Region, Prioritized Questions (Delphi round 2, n=44 questions)

**Table S5.** Setting Research Priorities for Advanced HIV Disease in Latin America and the Caribbean, Participant Characteristics (Delphi rounds 1 and 2)

**Table S1. Composition of the PAHO Regional Technical Advisory Group on Advanced HIV Disease in Latin America and the Caribbean**

| **Name** | **Affiliation** | **Country** | **Gender** | **Role** |
| --- | --- | --- | --- | --- |
| Freddy Perez | Pan American Health Organization (PAHO) | USA | Male | Coordinator |
| Omar Sued | Pan American Health Organization (PAHO) | USA | Male | HIV Advisor |
| Evelina Chapman | Pan American Health Organization (PAHO) | Chile | Female | Methods Advisor |
| Jorge Barreto | Pan American Health Organization (PAHO) | Brazil | Male | Methods Advisor |
| Antonio Camiro | Pan American Health Organization (PAHO) | México | Male | HIV Advisor |
| Ludovic Reveiz | Pan American Health Organization (PAHO) | USA | Male | Methods Advisor |
| Claudia Cortez | Facultad de Medicina, Universidad de Chile and Fundación Arriaran, Santiago, Chile | Chile | Female | HIV Advisor |
| Brenda Crabtree Ramírez | Departamento de Infectologia, Instituto Nacional de Ciencias Médicas y Nutrición Salvador Zubirán, México City, México. | México | Female | HIV Advisor |
| José Vidal | Departamento de Infectologia e Medicina Tropical, Hospital das Clínicas, Faculdade de Medicina da Universidade de São Paulo, São Paulo, Brasil | Brazil | Male | HIV Advisor |

**Table S2. PubMed Search Strategy for Systematic Reviews on Advanced HIV Disease (August 25, 2024)**

| **Search Component** | **Query** | **Records Retrieved** |
| --- | --- | --- |
| **HIV Terms** | ((("HIV Infections"[MeSH] OR "advanced HIV disease"[Title/Abstract] OR "low CD4 cells"[Title/Abstract]) AND "HIV Infections"[Title/Abstract]) OR "HIV Infection"[Title/Abstract] OR "htlv iii infections"[Title/Abstract] OR "HTLV-III Infection"[Title/Abstract] OR "htlv iii lav infections"[Title/Abstract] OR "HTLV-III-LAV Infection"[Title/Abstract] OR "HIV Coinfection"[Title/Abstract] OR "HIV Coinfections"[Title/Abstract] | – |
| **Virus-Specific Terms** | ("HIV"[MeSH] OR "HIV"[Title/Abstract] OR "HTLV-III"[Title/Abstract] OR "Human Immunodeficiency Virus"[Title/Abstract] OR "Human Immunodeficiency Viruses"[Title/Abstract] OR "human t cell lymphotropic virus type iii"[Title/Abstract] OR "human t cell leukemia virus type iii"[Title/Abstract] OR "LAV-HTLV-III"[Title/Abstract] OR "lymphadenopathy associated virus"[Title/Abstract] OR "AIDS Virus"[Title/Abstract] OR "Acquired Immunodeficiency Syndrome Virus"[Title/Abstract]) | – |
| **HIV-1/HIV-2 Terms** | ("hiv-1"[MeSH] OR "hiv-1"[Title/Abstract] OR "Human Immunodeficiency Virus Type 1"[Title/Abstract] OR "HIV-I"[Title/Abstract]) OR ("hiv-2"[MeSH] OR "hiv-2"[Title/Abstract] OR "HTLV-IV"[Title/Abstract] OR "Human Immunodeficiency Virus Type 2"[Title/Abstract]) | – |
| **STI Filter** | ("sexually transmitted diseases, viral"[MeSH] OR "Viral Sexually Transmitted Disease"[Title/Abstract]) | – |
| **Final Query** | Combined HIV terms AND Virus terms AND HIV-1/HIV-2 terms AND STI filter | 2,425 (SR) |
| **Limits** | (y_10[Filter] AND systematicreview[Filter]) | – |

**Table S3 Research Priorities for Advanced HIV Disease in Latin America and the Caribbean Region, Prioritized Questions (Survey 1, n=102 questions)**

| **No.** | **Group** | **Theme** | **Research Question** | **No. Answers** | **Score** |
| --- | --- | --- | --- | --- | --- |
| **High priority** | | | | | |
| 1 | Children | Frequency of Disease | What is the incidence and prevalence of opportunistic infections in children living with HIV in Latin America and the Caribbean ? | 8 | 0,95 |
| 2 | Children | Risk Factors | What factors contribute to the high rates of loss to follow-up among children under 5 living with HIV in Latin America and the Caribbean? | 7 | 0,94 |
| 3 | Adults | Frequency of Disease | What is the prevalence of histoplasmosis in individuals with advanced HIV (measured by antigenuria) in Latin America and the Caribbean? | 14 | 0,93 |
| 4 | Children | Mortality | What strategies are effective in ensuring retention during antiretroviral treatment for children living with HIV and minimizing premature mortality in Latin America and the Caribbean? | 7 | 0,92 |
| 5 | Children | Frequency of Disease | What is the rate and risk factors for mortality in children and adolescents with co-infection of HIV and tuberculosis in Latin America and the Caribbean? | 8 | 0,91 |
| 6 | Adults | Systems & Services | What are the barriers to early initiation of antiretroviral therapy in newly diagnosed individuals with advanced HIV disease? | 44 | 0,91 |
| 7 | Adults | Diagnosis, Prevention, Treatment, Prognosis | What is the impact on mortality of combined diagnostic interventions such as urine LAM, cryptococcal antigen tests, rapid CD4 counts, and viral load measurements in individuals on ARVs who are hospitalized in Latin America and the Caribbean? | 45 | 0,91 |
| 8 | Adults | Risk Factors | What are the most significant and modifiable risk factors contributing to tuberculosis-associated mortality in people living with HIV? | 47 | 0,90 |
| 9 | Children | Systems & Services | What are the effects of integrating HIV testing into immunization consultations for children to increase early diagnosis? | 7 | 0,89 |
| 10 | Adults | Policies | What comprehensive strategies can be developed to reduce treatment abandonment rates for tuberculosis in vulnerable groups with advanced HIV disease (e.g., homeless individuals, men-who-have-sex-with[men, etc.)? | 43 | 0,89 |
| 11 | Adults | Treatment & Therapy | In individuals living with HIV and co-infection of tuberculosis and histoplasmosis, what are the best available treatment options since the use of first-line therapies for both diseases (Itraconazole and H/R/P/E) is contraindicated concurrently? | 44 | 0,89 |
| 12 | Adults | Frequency of Disease | What is the prevalence of cryptococcal meningitis antigenemia in individuals living with HIV and CD4 <200/<100 in Latin America and the Caribbean? | 46 | 0,89 |
| 13 | Adults | Systems & Services | What are the barriers and facilitators for the different interventions aimed at improving linkage to health services for people with advanced HIV disease? For example, psychosocial interventions, use of e-health, etc. | 44 | 0,88 |
| 14 | Adults | Policies & Treatment | Are service integration models (e.g., HIV care, tuberculosis, substance abuse treatment, maternal-child transmission prevention programs, and mental health care for HIV) effective for retention or re-linkage to antiretroviral therapy in patients with advanced HIV disease? | 45 | 0,88 |
| 15 | Adults | Systems & Services | What interventions can be implemented to ensure proper linkage and continuity of support after hospital discharge for patients with advanced HIV disease to reduce mortality? | 45 | 0,88 |
| 16 | Adults | Systems & Services | What is the prevalence of late initiation of antiretroviral therapy in individuals with newly diagnosed advanced HIV disease in Latin America and the Caribbean? | 44 | 0,88 |
| 17 | Adults | Policies & Risk Factors | What gaps exist at different levels (patients, providers, health system) that contribute to loss to follow-up of patients living with HIV in Latin America and the Caribbean? | 43 | 0,88 |
| 18 | Adults | Diagnosis & Screening | What adherence assessment instruments for antiretroviral medications are effective in predicting the risk of treatment failure in individuals with advanced HIV disease? | 47 | 0,88 |
| 19 | Adults | Treatment & Therapy | What is the efficacy and safety of preventive treatment regimens for people living with HIV and positive cryptococcal antigenemia in plasma or peripheral blood in Latin America and the Caribbean? | 40 | 0,88 |
| 20 | Adults | Diagnosis & Screening | What is the impact of cryptococcal antigen detection (AgCr) and preventive therapy with fluconazole on the incidence of cryptococcal meningitis among adults living with HIV without prior antiretroviral treatment and with CD4 counts <100/μL? | 46 | 0,88 |
| 21 | Adults | Treatment & Therapy | What is the effectiveness and safety of the induction schemes used in the treatment of cryptococcal meningitis in individuals living with HIV in Latin America and the Caribbean? | 42 | 0,87 |
| 22 | Children | Systems & Services | What scalable strategies for community-based primary health interventions are effective in improving health outcomes related to HIV in the maternal-child population in the context of Latin America and the Caribbean? | 7 | 0,86 |
| 23 | Adults | Treatment | What is the impact of counseling for patients with advanced HIV disease and poor adherence to treatments to re-link initial antiretroviral therapy and achieve viral suppression? | 44 | 0,86 |
| 24 | Adults | Systems & Services | What are effective strategies to improve retention in prenatal and/or postpartum care among adolescents and young women living with HIV? | 43 | 0,86 |
| 25 | Adults | Frequency of Disease | What is the prevalence of Kaposi's sarcoma in individuals with advanced HIV in Latin America and the Caribbean? | 42 | 0,86 |
| 26 | Adults | Diagnosis & Screening | What is the performance of the concurrent use of Xpert in sputum and TB-LAM in urine for the diagnosis of Mycobacterium tuberculosis infection in the bloodstream in patients with advanced HIV disease? | 45 | 0,86 |
| 27 | Adults | Diagnosis & Screening | What is the cost-effectiveness of HIV-1 RNA tests at the point of care (POC) in the Latin America and the Caribbean region? | 47 | 0,85 |
| 28 | Adults | Prevention | What prevention and treatment strategies for HIV can be implemented in institutions with incarcerated individuals in Latin America and the Caribbean? | 44 | 0,85 |
| 29 | Adults | Systems & Services | What is the effect of patient navigation for individuals with advanced HIV across different population groups and outcomes in the HIV care continuum (e.g., re-linkage, retention)? | 42 | 0,85 |
| 30 | Adults | Diagnosis & Screening | Given that cervical cancer in women with HIV tends to present in more advanced forms compared to HIV-negative women, what are the strategies for diagnosis, treatment, and follow-up for both conditions that could improve quality of life and reduce mortality in Latin America and the Caribbean? | 16 | 0,85 |
| 31 | Adults | Treatment & Therapy | What is the comparative effectiveness and safety of liposomal amphotericin B versus desoxycholate amphotericin B and other antifungal treatments for primary disseminated histoplasmosis (PDH) in individuals living with HIV in Latin America and the Caribbean? | 43 | 0,85 |
| 32 | Adults | Systems & Services | Is the three-month treatment regimen with isoniazid and rifapentine, known as 3HP, cost-effective for preventing tuberculosis in patients with advanced HIV? | 41 | 0,85 |
| 33 | Children | Systems & Services | What interventions are effective in ensuring continuity of care for individuals living with HIV and preventing progression during the transition from pediatric to adult care in Latin America and the Caribbean? | 7 | 0,84 |
| 34 | Adults | Frequency of Disease | What is the prevalence of advanced HIV disease in adolescents who come from pediatric services in Latin America and the Caribbean? | 43 | 0,84 |
| 35 | Adults | Policies & Treatment | Are integrated psychosocial interventions in care programs for advanced HIV disease effective/cost-effective in reducing severe morbidity or long-term mortality in Latin America and the Caribbean? | 44 | 0,84 |
| 36 | Adults | Systems & Services | How can the experiences and perspectives of Indigenous peoples from Latin America and the Caribbean be integrated into HIV care to improve access to culturally adapted and safe healthcare? | 43 | 0,84 |
| 37 | Children | Diagnosis & Screening | What interventions are effective for early diagnosis of HIV infection in infants exposed to the virus during the first 4-8 weeks of life? | 7 | 0,83 |
| 38 | Children | Diagnosis & Screening | What factors influence the cost-effectiveness relationship of point-of-care HIV testing compared to standard HIV screening tests in children under 18 months? | 7 | 0,83 |
| 39 | Children | Diagnosis & Screening | What are the optimal strategies for tuberculosis screening in children living with HIV in Latin America and the Caribbean? | 8 | 0,83 |
| 40 | Adults | Policies & Risk Factors | What sociodemographic variables (racial and ethnic minorities) influence health literacy in people living with HIV, and how do they affect adherence to antiretroviral treatment and utilization of health services? | 42 | 0,83 |
| 41 | Adults | Frequency of Disease | What is the prevalence of lymphoproliferative diseases, including primary central nervous system lymphoma, in patients with advanced HIV in Latin America and the Caribbean? | 15 | 0,83 |
| 42 | Adults | Treatment & Therapy | What are the factors for an increased risk of acquired resistance to rifamycin as the first-line treatment for TB in individuals with advanced HIV? | 39 | 0,83 |
| 43 | Adults | Systems & Services | What role do community, virtual, and differentiated service provision models play in addressing the comorbidity of tuberculosis and HIV in Latin America and the Caribbean? | 40 | 0,83 |
| 44 | Adults | Frequency of Disease | What is the burden of cytomegalovirus disease in patients with advanced HIV in Latin America and the Caribbean? | 44 | 0,83 |
| 45 | Children | Policies | How can adherence to antiretroviral treatment in children and adolescents living with HIV, especially among those who are double orphans, be improved? | 7 | 0,82 |
| 46 | Children | Diagnosis & Screening | What is the cost-effectiveness of modern tuberculosis diagnostic tests (such as FujiLAM, AlereLAM, IGRA) to detect co-infection in children living with HIV? | 8 | 0,82 |
| 47 | Adults | Frequency of Disease | What is the frequency of reactivation of Toxoplasma gondii in individuals living with HIV in Latin America and the Caribbean? | 44 | 0,82 |
| 48 | Adults | Risk Factors | What is the role of advanced HIV disease in therapeutic failures of multi-drug-resistant tuberculosis (TB)? | 45 | 0,82 |
| 49 | Adults | Systems & Services | Does the combination of reminder/alarm devices with other strategies, such as counseling, result in cost-effective and acceptable improvements in medication adherence among pregnant women living with HIV in Latin America and the Caribbean? | 42 | 0,81 |
| 50 | Adults | Systems & Services | What is the effectiveness of text messages via mobile phones to improve adherence to antiretroviral therapy in adolescents in Latin America and the Caribbean? | 43 | 0,81 |
| 51 | Adults | Diagnosis & Screening | What would be the best strategies for clinical suspicion and screening for Mycobacterium avium-intracellulare complex (MAC) disease in individuals living with HIV? | 44 | 0,81 |
| 52 | Adults | Treatment & Therapy | How does the effectiveness and safety of preventive treatment with isoniazid for tuberculosis differ among various subpopulations (with different health determinants) of individuals living with HIV in Latin America and the Caribbean? | 40 | 0,81 |
| 53 | Adults | Policies & Treatment | How does the effectiveness of contingency management vary by different demographic groups and substance use types to improve adherence to antiretroviral therapy among people living with HIV? | 40 | 0,80 |
| 54 | Children | Follow-up | What is the impact of the transition stage from pediatric to adult care on the continuity of HIV care in Latin America and the Caribbean? | 6 | 0,80 |
| 55 | Adults | Frequency of Disease | What is the frequency of Mycobacterium avium-intracellulare complex disease in individuals with advanced HIV? | 44 | 0,80 |
| 56 | Adults | Diagnosis & Screening | What is the performance of advanced versions of the QuantiFERON-TB (QFT) | 40 | 0,80 |
| 57 | Adults | Policies | What interventions can improve health outcomes for women living with advanced HIV during and after incarceration? | 42 | 0,80 |
| 58 | Adults | Diagnosis & Screening | What is the performance of advanced versions of the QuantiFERON-TB (QFT-plus) test for detecting latent tuberculosis infection (LTBI) in patients with advanced HIV disease? | 40 | 0,80 |
| **Medium priority** | | | | | |
| 59 | Children | Prevention | What is the effectiveness, safety, and immunity following vaccination in children living with HIV who are on ARV treatment? | 7 | 0,79 |
| 60 | Adults | Prognosis | What is the prognosis of cytomegalovirus disease in patients with advanced HIV in Latin America and the Caribbean? | 40 | 0,79 |
| 61 | Adults | Treatment & Therapy | What are the clinical outcomes and efficacy of amphotericin B treatment in pregnant women living with HIV with cryptococcal meningitis? | 42 | 0,79 |
| 62 | Adults | Mortality | What is the impact of weight loss on mortality in individuals living with HIV, who are hospitalized and receiving antiretroviral therapy? | 45 | 0,79 |
| 63 | Children | Systems & Services | How can prospective evaluations improve the implementation of detection strategies in children living with HIV in resource-limited settings? | 7 | 0,78 |
| 64 | Children | Risk Factors | Is childhood sexual abuse a risk factor for non-adherence to treatment in children with HIV? | 7 | 0,77 |
| 65 | Adults | Policies & Risk Factors | Is there an association between food insecurity and CD4 count among individuals living with HIV? | 43 | 0,78 |
| 66 | Adults | Screening & Risk Factors | In women living with HIV and with cervical intraepithelial neoplasia that is not adequately controlled with antiretroviral treatment, how often should they be monitored for high-grade cervical intraepithelial neoplasia (CIN2+)? | 16 | 0,78 |
| 67 | Adults | Treatment | What is the effectiveness of different existing treatments for Kaposi's sarcoma in individuals living with HIV? | 16 | 0,78 |
| 68 | Children | Diagnosis & Screening | What is the availability and access to rapid diagnostic tests for tuberculosis in children with advanced HIV? | 8 | 0,77 |
| 69 | Children | Policies | How can prospective evaluations improve the implementation of tuberculosis screening strategies in children living with HIV in Latin America and the Caribbean? | 8 | 0,77 |
| 70 | Children | Prognosis | Does childhood sexual abuse, as a risk factor for non-adherence to treatment in children living with HIV, impact mortality? | 7 | 0,77 |
| 71 | Adults | Prognosis | What is the prognostic value of weight loss regarding mortality in hospitalized patients with HIV treated with antiretrovirals in Latin America and the Caribbean? | 43 | 0,77 |
| 72 | Adults | Diagnosis & Prognosis | Will identifying HPV genotypes associated with high-grade cervical disease in women living with HIV improve selective screening and timely treatment of cervical disease in Latin America and the Caribbean? | 16 | 0,77 |
| 73 | Adults | Prevention | What effective and safe vaccination strategies for each existing vaccine would improve humoral immune response in adults with advanced HIV disease? | 42 | 0,77 |
| 74 | Children | Diagnosis & Screening | What strategies are effective for implementing and sustaining long-term HIV screening programs in pediatric emergency services in Latin America and the Caribbean? | 7 | 0,76 |
| 75 | Adults | Frequency of Disease | What is the prevalence of opportunistic germ-related diarrhea (e.g., Cryptosporidium) in patients with advanced HIV in Latin America and the Caribbean? | 44 | 0,76 |
| 76 | Adults | Treatment & Therapy | What is the most effective systemic antifungal therapy for treating sporotrichosis in individuals with advanced HIV? | 36 | 0,76 |
| 77 | Adults | Treatment & Therapy | What is the most effective and safe therapy to prevent end-organ disease and associated mortality in patients with advanced HIV co-infected with cytomegalovirus? | 43 | 0,75 |
| 78 | Children | Diagnosis & Screening | What is the magnitude of dropout rates from early diagnostic services in children exposed to HIV in Latin America and the Caribbean? | 7 | 0,74 |
| 79 | Adults | Mortality | Could machine learning be an effective method for predicting deaths among individuals living with HIV in Latin America and the Caribbean? | 44 | 0,74 |
| 80 | Adults | Treatment & Therapy | Should anti-CMV therapy be administered to individuals with advanced HIV and positive PCR for CMV in the absence of end-organ disease? | 44 | 0,74 |
| 81 | Adults | Prevention | What is the effectiveness and safety of the Mpox vaccine in patients with advanced HIV? | 40 | 0,74 |
| 82 | Adults | Frequency of Disease | What is the prevalence of ocular toxoplasmosis among individuals living with advanced HIV in Latin America and the Caribbean? | 45 | 0,73 |
| 83 | Adults | Frequency of Disease | What is the prevalence of co-infection with malaria and HIV in Latin America and the Caribbean? | 39 | 0,73 |
| 84 | Adults | Frequency of Disease | What is the prevalence of visceral leishmaniasis in individuals with advanced HIV in LAC? | 38 | 0,73 |
| 85 | Children | Diagnosis & Screening | What is the sensitivity and specificity of modern tuberculosis diagnostic tests (such as FujiLAM and AlereLAM) for detecting co-infection in children living with HIV? | 8 | 0,72 |
| 86 | Adults | Prognosis | How do the immunological and virological characteristics of individuals living with HIV affect the clinical manifestations and severity of mpox? | 39 | 0,72 |
| 87 | Adults | Diagnosis & Screening | What is the accuracy of traditional fundoscopy and new AI tools for diagnosing ocular toxoplasmosis in individuals with advanced HIV? | 44 | 0,72 |
| 88 | Adults | Diagnosis & Screening | Does the CD4 T-cell count affect the accuracy of the interferon-gamma release assay (IGRA) for diagnosing TB in individuals living with HIV? | 43 | 0,72 |
| **Low Priority** | | | | | |
| 89 | Adults | Diagnosis, Prevention, Treatment, Prognosis | What are the best strategies for diagnosis, prophylaxis, complete treatment (first and second therapy), and follow-up regarding cure, relapse, and mortality in patients with HIV and co-infection by human visceral leishmaniasis? | 41 | 0,69 |
| 90 | Adults | Frequency of Disease | What is the frequency of co-infection by sporotrichosis in individuals with advanced HIV in Latin America and the Caribbean? | 42 | 0,69 |
| 91 | Adults | Frequency of Disease | What is the frequency of hepatic tuberculosis as a co-infection in individuals with advanced HIV in Latin America and the Caribbean? | 42 | 0,69 |
| 92 | Adults | Diagnosis & Screening | What are the epidemiological, clinical, and laboratory aspects associated with co-infection of human visceral leishmaniasis and HIV? | 42 | 0,68 |
| 93 | Adults | Diagnosis & Screening | Are diagnostic methods and staging available for Kaposi's sarcoma in individuals with advanced HIV in Latin America and the Caribbean? | 16 | 0,68 |
| 94 | Adults | Prognosis | What factors influence the severity and worst clinical outcome of co-infection by sporotrichosis in individuals with advanced HIV? | 39 | 0,68 |
| 95 | Children | Diagnosis & Screening | What is the most appropriate time to integrate HIV testing at birth, initiation of antiretroviral therapy, and the timing of BCG vaccination? | 8 | 0,67 |
| 96 | Adults | Frequency of Disease | What is the prevalence of sexually transmitted intestinal spirochetosis in MSM living with advanced HIV in Latin America and the Caribbean? | 40 | 0,65 |
| 97 | Adults | Prognosis | How does the HIV serostatus and the presence of CD4 <200 affect the clinical course and mortality from infective endocarditis? | 39 | 0,65 |
| 98 | Adults | Frequency of Disease | Is it relevant to know the prevalence of methicillin-resistant Staphylococcus aureus (MRSA) colonization in individuals living with HIV at high risk for disease progression (e.g., incarcerated individuals) in Latin America and the Caribbean? | 42 | 0,65 |
| 99 | Adults | Diagnosis & Screening | Is it necessary to routinely measure vitamin D levels and provide permanent vitamin D supplementation in individuals with advanced HIV in Latin America and the Caribbean? | 45 | 0,63 |
| 100 | Children | Treatment | What is the effectiveness of different antiretroviral therapy regimens, including high-dose vitamin A, to reduce the risk of postnatal HIV transmission? | 7 | 0,58 |
| 101 | Children | Treatment | What would be the effective and safe dosage of probiotics as a complement to routine antiretroviral therapy to decrease CD4+ T-cell counts? | 5 | 0,52 |
| 102 | Children | Treatment | Does the use of probiotics as a complement to routine antiretroviral therapy in children living with HIV, in addition to changes in CD4+ T-cell counts, provide long-term clinical improvement? | 6 | 0,47 |

**Table S4. Research Priorities for Advanced HIV Disease in Latin America and the Caribbean Region, Prioritized Questions (Survey 2, n=44 questions)**

| **No.** | **Group** | **Theme** | **Research Question** | **No. Answers** | **Score** |
| --- | --- | --- | --- | --- | --- |
| **High Priority** |  |  |  |  |  |
| **1** | **Adults** | **Polices** | In medium/low-resource settings, is it better to centralize or decentralize laboratories with demands for diagnostic tests for opportunistic infections in individuals with advanced HIV? | 61 | 0,9 |
| **2** | **Children** | **Systems / Services** | What is the availability and access to rapid diagnostic tests for tuberculosis in children with advanced HIV? | 9 | 0,89 |
| **3** | **Adults** | **Frequency of Disease** | What is the prevalence of cryptococcosis in individuals with advanced HIV? | 61 | 0,89 |
| **4** | **Adults** | **Risk Factors** | Will identifying HPV genotypes associated with high-grade cervical disease in women with HIV improve selective screening and timely treatment of cervical disease in LAC? | 59 | 0,85 |
| **5** | **Adults** | **Risk Factors** | What is the impact of poverty and deprivation on disease progression and death in individuals with advanced HIV? | 60 | 0,85 |
| **6** | **Children** | **Systems / Services** | What is the magnitude of dropout rates from early diagnostic services in children exposed to HIV in Latin America and the Caribbean? | 9 | 0,83 |
| **7** | **Adults** | **Prevention** | What is the impact of the HPV vaccine in individuals with advanced HIV and the presence of HPV-related lesions? | 59 | 0,83 |
| **8** | **Adults** | **Diagnosis** | Is it cost-effective to include a rapid molecular test in saliva at the point of care for managing pneumocystosis in individuals with advanced HIV? | 62 | 0,82 |
| **9** | **Adults** | **Frequency of Disesase** | What is the prevalence of disseminated histoplasmosis in adolescents and adults with advanced HIV? | 61 | 0,8 |
| **10** | **Adults** | **Policies** | What is the impact of peer support programs for women on linking to care after diagnosis at late stages of the disease and on retention in care among newly diagnosed cisgender women? | 60 | 0,8 |
| **Medium Priority** |  |  |  |  |  |
| **11** | **Children** | **Frequency of Disease** | What is the prevalence of disseminated histoplasmosis in children under 5 years old? | 9 | 0,78 |
| **12** | **Adults** | **Frequency of Disease** | What is the prevalence of advanced HIV disease in transgender individuals and their health outcomes compared to other groups? | 60 | 0,78 |
| **13** | **Adults** | **Diagnosis** | Is self-testing effective in preventing late diagnosis of advanced HIV disease in migrants? | 61 | 0,77 |
| **14** | **Adults** | **Treatment** | What is the effectiveness of different existing treatments for Kaposi's sarcoma in individuals living with HIV? | 58 | 0,76 |
| **15** | **Adults** | **Prevention** | What effective and safe vaccination strategies for each existing vaccine would improve humoral immune response in adults with advanced HIV disease? | 60 | 0,76 |
| **16** | **Adults** | **Risk Factors** | What are the differences in the natural history of HPV infection between individuals with advanced HIV vs those without advanced disease? | 60 | 0,76 |
| **17** | **Adults** | **Management** | In women with HIV and cervical intraepithelial neoplasia that is not adequately controlled with antiretroviral treatment, how often should they be monitored for high-grade cervical intraepithelial neoplasia (CIN2+)? | 59 | 0,76 |
| **18** | **Adults** | **Diagnosis** | What is the performance of PCR in blood for disseminated histoplasmosis in individuals with advanced HIV? | 58 | 0,76 |
| **19** | **Children** | **Policies** | What strategies are effective for implementing and sustaining long-term HIV screening programs in pediatric emergency services in Latin America and the Caribbean? | 8 | 0,75 |
| **20** | **Adults** | **Risk Factors** | How do the immunological and virological characteristics of individuals living with HIV affect the clinical manifestations and severity of Mpox? | 60 | 0,74 |
| **21** | **Adults** | **Prevention** | What is the effectiveness and safety of the Mpox vaccine in patients with advanced HIV? | 59 | 0,73 |
| **22** | **Children** | **Policies** | How can prospective evaluations improve the implementation of tuberculosis screening strategies in children living with HIV in LAC? | 7 | 0,71 |
| **23** | **Adults** | **Diagnosis** | Does the CD4 T-cell count affect the accuracy of the interferon-gamma release assay (IGRA) for diagnosing TB in individuals living with HIV? | 59 | 0,71 |
| **24** | **Adults** | **Risk Factors** | Is there an association between food insecurity and CD4 count among individuals living with HIV in Latin America and the Caribbean? | 59 | 0,68 |
| **25** | **Children** | **Diagnosis** | What is the sensitivity and specificity of modern tuberculosis diagnostic tests (such as FujiLAM and AlereLAM) for detecting co-infection in children living with HIV? | 9 | 0,67 |
| **26** | **Adults** | **Prognosis** | What is the prognosis of cytomegalovirus disease in patients with advanced HIV in Latin America and the Caribbean? | 59 | 0,65 |
| **27** | **Children** | **Prevention** | What is the effectiveness, safety, and post-administration immunity of different vaccines in children living with HIV who are on ARV treatment? | 9 | 0,64 |
| **28** | **Adults** | **Prevention** | Is preventive treatment for cytomegalovirus disease necessary in patients with advanced HIV? | 59 | 0,64 |
| **29** | **Adults** | **Risk Factors** | What is the association between alcohol consumption and mortality in individuals with advanced HIV? | 60 | 0,63 |
| **30** | **Adults** | **Diagnosis** | What is the accuracy of traditional fundoscopy and new AI tools for diagnosing ocular toxoplasmosis in individuals living with HIV? | 59 | 0,61 |
| **31** | **Adults** | **Prognosis** | What is the prognostic value of body weight (or BMI) at the time of diagnosis of advanced HIV disease in adults? | 59 | 0,61 |
| **32** | **Adults** | **Risk Factors** | Is there an association between Chemsex and abandonment of antiretroviral therapy in individuals with advanced HIV? | 60 | 0,61 |
| **33** | **Adults** | **Frequency of Disease** | What is the prevalence of opportunistic germ-related diarrhea (e.g., Cryptosporidium) in individuals with advanced HIV in Latin America and the Caribbean? | 59 | 0,6 |
| **Low Priority** |  |  |  |  |  |
| **34** | **Adults** | **Policies** | What are the benefits of economic incentive programs for individuals with advanced HIV? | 59 | 0,59 |
| **35** | **Adults** | **Frequency of Disease** | What is the prevalence of coccidioidomycosis in individuals with advanced HIV in areas where it is endemic? | 58 | 0,59 |
| **36** | **Adults** | **Mortality** | Could machine learning be an effective method for predicting deaths among individuals living with HIV in Latin America and the Caribbean? | 61 | 0,58 |
| **37** | **Adults** | **Frequency of Disease** | What is the prevalence of ocular toxoplasmosis among individuals with advanced HIV in Latin America and the Caribbean? | 59 | 0,58 |
| **38** | **Adults** | **Frequency of Disease** | What is the prevalence of adult T-cell leukemia lymphoma in individuals with advanced HIV in Latin America and the Caribbean? | 58 | 0,58 |
| **39** | **Adults** | **Treatment** | What is the most effective systemic antifungal therapy for treating sporotrichosis in individuals with advanced HIV? | 59 | 0,56 |
| **40** | **Children** | **Risk Factors** | Is childhood sexual abuse a risk factor for non-adherence to treatment in children with HIV? | 8 | 0,53 |
| **41** | **Adults** | **Treatment** | What are the best strategies for diagnosis, prophylaxis, complete treatment (first and second therapies), and follow-up, in terms of cure, relapse, and mortality in patients with HIV and co-infection by human visceral leishmaniasis? | 57 | 0,48 |
| **42** | **Adults** | **Frequency of Disease** | What is the prevalence of co-infection with malaria and HIV in individuals with advanced HIV in Latin America and the Caribbean? | 58 | 0,44 |
| **43** | **Children** | **Risk Factors** | Would childhood sexual abuse, as a risk factor for non-adherence to treatment in children living with HIV, impact mortality? | 8 | 0,41 |
| **44** | **Adults** | **Frequency of Disease** | What is the prevalence of visceral leishmaniasis in individuals with advanced HIV in Latin America and the Caribbean? | 59 | 0,35 |

**Table S5. Setting Research Priorities for Advanced HIV Disease in Latin America and the Caribbean, Participant Characteristics (Delphi rounds 1 and 2)**

|  | Round 1 | | Round 2 | | p-value* |
| --- | --- | --- | --- | --- | --- |
| *Country* | **n** | **%** | **n** | **%** | 0.984 |
| Argentina | 8 | 10.7% | 4 | 5.8% |  |
| Bolivia | 3 | 4.0% | 4 | 5.8% |  |
| Brazil | 13 | 17.3% | 11 | 15.9% |  |
| Chile | 7 | 9.3% | 8 | 11.6% |  |
| Colombia | 3 | 4.0% | 4 | 5.8% |  |
| Costa Rica | 1 | 1.3% | 2 | 2.9% |  |
| Dominican Republic | 1 | 1.3% | 3 | 4.3% |  |
| Honduras | 1 | 1.3% | 0 | 0.0% |  |
| El Salvador | 2 | 2.7% | 3 | 4.3% |  |
| Guatemala | 2 | 2.7% | 2 | 2.9% |  |
| Mexico | 15 | 20.0% | 8 | 11.6% |  |
| Panama | 1 | 1.3% | 1 | 1.4% |  |
| Paraguay | 1 | 1.3% | 1 | 1.4% |  |
| Peru | 5 | 6.7% | 8 | 11.6% |  |
| Trinidad & Tobago | 3 | 4.0% | 2 | 2.9% |  |
| Uruguay | 1 | 1.3% | 1 | 1.4% |  |
| USA | 1 | 1.3% | 1 | 1.4% |  |
| Venezuela | 2 | 2.7% | 3 | 4.3% |  |
| Not Reported | 5 | 6.7% | 3 | 4.3% |  |
| *Occupation* |  |  |  |  | 0.952 |
| Academic or Educator | 8 | 10.8% | 7 | 10.1% |  |
| Activist/Community Leader | 3 | 4.1% | 2 | 2.9% |  |
| Epidemiologist | 1 | 1.4% | 1 | 1.4% |  |
| Program and NGO Manager | 5 | 6.8% | 4 | 5.8% |  |
| Researcher | 21 | 28.4% | 14 | 20.3% |  |
| Physician | 28 | 37.8% | 30 | 43.5% |  |
| Other Health Professional | 5 | 6.8% | 7 | 10.1% |  |
| Policy Maker | 3 | 4.1% | 4 | 5.8% |  |
| *Organization* |  |  |  |  | 0.974 |
| UN Agency | 2 | 2.7% | 1 | 1.4% |  |
| Foundation | 1 | 1.4% | 1 | 1.4% |  |
| National, Regional, or Local Government | 22 | 29.7% | 17 | 24.6% |  |
| Hospital/Clinic | 23 | 31.1% | 25 | 36.2% |  |
| Academic/Research Institution | 15 | 20.3% | 16 | 23.2% |  |
| International Organization (Not UN-affiliated) | 1 | 1.4% | 1 | 1.4% |  |
| NGO | 10 | 13.5% | 8 | 11.6% |  |
| *Age* |  |  |  |  | 0.834 |
| 25-34 | 2 | 2.7% | 4 | 5.8% |  |
| 35-44 | 29 | 39.2% | 26 | 37.7% |  |
| 45-54 | 26 | 35.1% | 25 | 36.2% |  |
| 55-64 | 12 | 16.2% | 8 | 11.6% |  |
| 65+ | 5 | 6.8% | 6 | 8.7% |  |
| *Gender* |  |  |  |  | 1 |
| Cisgender Man | 40 | 54.8% | 38 | 55.1% |  |
| Cisgender Woman | *33* | *45.2%* | *31* | *44.9%* |  |
| Non-binary | 1 | 1.4% | 0 | 0.0% |  |

*Fisher's exact test. NGO= Non-Governmental Organization; UN=United Nations
